# Oral Administration of *Aster yomena* Butanol Fraction Attenuates DNCB-Induced Atopic Dermatitis-like Skin Inflammation in Mice: Implications as a Dietary Supplement Candidate for Companion Animals

**DOI:** 10.3390/ani16132003

**Published:** 2026-06-30

**Authors:** Hyeon Jeong Moon, Chung-Do Lee, Seon-Jin Lee, Gun Lee, Jaewoo Choi, Geon Woo Kim, Jun Bong Lee, Jun-Gyu Park, Yeong-Bin Baek, Sang-Ik Park

**Affiliations:** 1 Department of Veterinary Pathology, College of Veterinary Medicine, Chonnam National University, Gwangju 61186, Republic of Korea; dals404@naver.com (H.J.M.); e05311cht@naver.com (J.C.); 2 Department of Veterinary Pathology, College of Veterinary Medicine and BK21 FOUR Program, Chonnam National University, Gwangju 61186, Republic of Korea; cndehsla@gmail.com (C.-D.L.); udlrjs77@naver.com (G.L.); 3 Department of Integrative Food, Bioscience and Biotechnology, Graduate School of Chonnam National University, Gwangju 61186, Republic of Korea; sunjin0011111@gmail.com; 4 Twell Co., Ltd., Yeongcheon 38882, Republic of Korea; ceo@twell.kr; 5 Department of Food and Environmental Hygiene, College of Veterinary Medicine, Chonnam National University, Gwangju 61186, Republic of Korea; jblee12@jnu.ac.kr; 6 Department of Veterinary Zoonotic Diseases, College of Veterinary Medicine, Chonnam National University, Gwangju 61186, Republic of Korea; kingsalt@jnu.ac.kr

**Keywords:** *Aster yomena*, atopic dermatitis, dietary supplement, functional feed additive, companion animals, DNCB-induced mouse model, rutin, dicaffeoylquinic acid, type 2 inflammation, skin health

## Abstract

Atopic dermatitis (AD) is a chronic inflammatory skin disease requiring long-term management in companion animals. This study evaluated the butanol fraction of *Aster yomena* extract (AY BuOH) as an orally administered dietary supplement candidate for atopic skin health using a DNCB-induced AD-like mouse model. AY BuOH was chemically profiled by UPLC-ESI-QToF-MS/MS and quantified for major marker compounds. Male SKH-1 mice were allocated to normal control, DNCB control, oclacitinib-treated, or AY BuOH-treated groups (2.5–640 mg/kg/day) for five weeks. AY BuOH contained abundant rutin and dicaffeoylquinic acid (DCQA) isomers, including 3,4-, 3,5-, and 4,5-DCQA isomers. Oral AY BuOH administration, particularly at intermediate doses, reduced modified dermatitis severity scores, gross skin lesions, serum immunoglobulin E levels, and cutaneous interleukin-4 and interleukin-13 concentrations, with effects comparable to those observed in the oclacitinib reference group in selected endpoints. AY BuOH also partially restored splenic Th1/Th2 balance and attenuated epidermal and dermal thickening. The highest dose, 640 mg/kg/day, was associated with body weight loss and elevated hepatic enzyme activities, suggesting potential hepatic stress. These findings provide preclinical evidence that AY BuOH may serve as a standardized plant-derived dietary supplement or functional feed additive candidate for companion animal skin health, pending target-species safety and efficacy validation.

## Simple Summary

Atopic dermatitis is a long-lasting allergic skin disease that causes itching, redness, swelling, and skin damage in both people and companion animals. Because this disease often needs repeated or long-term care, safe dietary ingredients that can support skin health are of interest. In this study, we tested a concentrated extract from *Aster yomena*, a plant traditionally used for inflammatory conditions, in mice with experimentally induced atopic dermatitis-like skin disease. Oral administration of the extract improved visible skin lesions, reduced allergy-related blood markers, lowered inflammatory signals in the skin, and improved microscopic skin changes. The most consistent effects were observed at intermediate doses, whereas the highest dose caused signs of liver stress. These findings suggest that *Aster yomena* butanol fraction may be a useful candidate ingredient for developing dietary supplements or functional feed additives aimed at supporting skin health in companion animals. Further studies in dogs and cats are needed before practical use.

## 1. Introduction

Atopic dermatitis (AD) is a chronic, relapsing inflammatory skin disorder that affects both humans and animals, characterized by intense pruritus, skin barrier dysfunction, and a dysregulated immune response. Its prevalence has been increasing globally in companion animals such as dogs as well as in humans, raising concerns regarding animal health, welfare, and their interactions with the environment [1]. In companion animals, particularly dogs, AD is one of the most common chronic inflammatory dermatoses and is closely associated with impaired quality of life, recurrent pruritus, secondary skin infections, and long-term management burdens for owners and veterinarians [2,3]. AD is a multifactorial disease involving genetic predisposition, epidermal barrier disruption, immune imbalance, microbial dysbiosis, and environmental triggers. Its pathophysiology is largely associated with excessive type 2 immune activation, elevated immunoglobulin E (IgE), and increased production of cytokines such as interleukin (IL)-4, IL-5, and IL-13, which contribute to chronic inflammation, pruritic skin lesions, and barrier impairment [4].

Conventional management strategies for canine AD include glucocorticoids, cyclosporine, allergen-specific immunotherapy, Janus kinase inhibitors such as oclacitinib, and other emerging therapeutic approaches that continue to be developed for canine atopic dermatitis [5,6,7]. These interventions are clinically effective, particularly for controlling pruritus and inflammation, but AD usually requires long-term or repeated management rather than short-term curative treatment [5,6]. Therefore, there is increasing interest in adjunctive nutritional strategies that can support skin barrier integrity, modulate inflammatory responses, and reduce the long-term reliance on pharmacological intervention. In this context, dietary supplements and functional feed additives should not be regarded as direct replacements for approved drugs, but rather as complementary approaches for maintaining skin health and improving resilience against chronic inflammatory stimuli in companion animals [8,9].

Plant-derived nutraceuticals and functional feed ingredients have attracted attention in companion animal nutrition because they provide bioactive compounds that can be incorporated into daily diets or supplement formulations. Unlike essential nutrients, many plant-derived compounds, including phenolic acids, flavonoids, terpenoids, and saponins, act as non-nutrient functional constituents with antioxidant, anti-inflammatory, immunomodulatory, and barrier-supportive activities [10]. This is particularly relevant in companion animals, where long-term owner adherence, palatability, safety, and compatibility with routine feeding are critical determinants of practical clinical utility [10,11]. Accordingly, plant-derived feed additives may offer a rational platform for developing non-pharmacological products aimed at supporting dermatological health, provided that their composition, effective dose range, and safety margins are adequately characterized.

Evidence supporting nutritional and functional-feed approaches in canine AD is gradually expanding. Essential fatty acids and skin-supportive diets have been investigated for their potential to improve epidermal lipid composition, skin barrier function, and clinical signs in atopic dogs [9,12]. More recently, a functional canine diet supplemented with a standardized plant-derived extract was reported to improve transepidermal water loss, lesion severity, and pruritus scores in dogs with AD, supporting the concept that diet-delivered functional ingredients can serve as adjunctive tools for managing chronic allergic skin disease in companion animals [8]. However, the number of plant-derived functional ingredients evaluated through integrated phytochemical, immunological, histopathological, and safety-oriented approaches remain limited. Such integrative preclinical studies are needed before candidate ingredients can be rationally advanced into target-species feeding trials.

To investigate AD-related mechanisms and screen candidate functional ingredients, murine models remain valuable preclinical tools. Among them, the 2,4-dinitrochlorobenzene (DNCB)-induced AD-like mouse model is widely used because repeated hapten exposure induces erythema, excoriation, lichenification, epidermal hyperplasia, inflammatory cell infiltration, increased IgE production, and type 2-skewed immune responses [13,14]. Although this model cannot fully reproduce spontaneous canine or feline AD, it provides a controlled platform for dose-ranging, mechanistic evaluation, and preliminary safety assessment before conducting formulation, palatability, pharmacokinetic, and efficacy studies in dogs or cats [14]. Therefore, findings from DNCB-induced mice should be interpreted as preclinical proof-of-concept evidence rather than direct clinical evidence for companion animals.

*Aster yomena* (AY), commonly known as Korean starwort, is a perennial herb traditionally used in East Asian medicine for inflammatory conditions [15,16]. Phytochemical studies have shown that AY contains abundant phenolic acids and flavonoids with antioxidant and anti-inflammatory potential. In vitro, AY extracts suppress nitric oxide production and downregulate NF-κB signaling in lipopolysaccharide-stimulated macrophages, suggesting immunomodulatory activity [15]. These properties make AY a plausible source of functional feed ingredients for inflammatory skin health, particularly when its active fractions can be chemically standardized and orally administered.

The butanol fraction of AY extract (AY BuOH) is enriched in phenolic and flavonoid compounds, including rutin, isoquercitrin, miquelianin, chlorogenic acid, and multiple dicaffeoylquinic acid (DCQA) derivatives such as 3,4-DCQA, 3,5-DCQA, and 4,5-DCQA [17]. These compounds are biologically relevant because caffeoylquinic acid derivatives and flavonoids have been associated with antioxidant, anti-inflammatory, anti-allergic, and skin-barrier-supportive effects in experimental inflammatory skin models [18,19]. From a feed-additive perspective, the presence of quantifiable marker compounds is important because it allows future product standardization, batch-to-batch quality control, and dose translation for companion animal applications.

In this study, we evaluated AY BuOH as an orally administered dietary supplement and functional feed additive candidate for atopic skin health using a DNCB-induced AD-like mouse model. Rather than assessing AY BuOH as a topical drug or direct pharmacological substitute, we focused on its potential as a standardized functional ingredient that can be chemically characterized, orally administered, and further developed for companion animal skin-health applications. By integrating phytochemical profiling, clinical lesion scoring, allergic inflammatory markers, immune-balance analysis, histopathology, and preliminary biochemical safety screening, this study provides preclinical evidence to guide future target-species validation of AY BuOH.

## 2. Materials and Methods

### 2.1. Sample Extraction and Solvent Fractionation

The aerial parts of AY were collected from Gurye, Republic of Korea. The collected samples were washed, air-dried under shade at room temperature, and ground into a fine powder using a blender before extraction. A total of 100 g of the dried powder was extracted with 2 L of distilled water at 95 °C for 30 min. The resulting hot-water extract was filtered through filter paper (No. 2, Advantec Toyo Kaisha, Tokyo, Japan) and partitioned three times with water-saturated n-butanol (BuOH; 2 L each time). The BuOH fraction was concentrated under reduced pressure at 38 °C using a rotary vacuum evaporator (N-1300, Eyela, Shanghai, China) until the solvent was completely removed. The remaining concentrate was subsequently lyophilized using a freeze-dryer (MLB-9003, Mareuda, Gwangju, Republic of Korea) to yield 5.3 g of dried BuOH fraction. The final extraction yield was 5.3% (*w*/*w*) based on the dried starting material. The dried BuOH fraction was stored at −20 °C until use.

### 2.2. UPLC-ESI-QToF-MS/MS Qualitative Analysis

Qualitative analysis was conducted using a quadrupole time-of-flight mass spectrometer (QToF-MS; Xevo G2-XS QToF, Waters, Manchester, UK) equipped with an ultra-performance liquid chromatography (UPLC; Acquity UPLC system, Waters, Milford, MA, USA) and electrospray ionization (ESI) source. Separation was performed on an Acquity UPLC HSS T3 column (1.8 μm, 2.1 × 100 mm, Waters) maintained at 40 °C. The mobile phase was composed of H2O containing 0.1% formic acid (solvent A) and MeCN containing 0.1% formic acid (solvent B). The gradient program was as follows: 5% B (0 min) → 5% B (2 min) → 10% B (5 min) → 25% B (15 min) → 30% B (24 min). Flow rate was 0.35 mL/min and injection volume was 1 μL. The MS conditions were as follows: scan range, *m*/*z* 50~1200; scan time, 0.2 s; ionization mode, negative; capillary voltage, 2.5 kV; sampling cone voltage, 40 V; cone gas flow, 50 L/h; desolvation gas flow, 800 L/h; desolvation temperature, 400 °C; ion source temperature, 130 °C; collision energy, 6 eV (low) and 25~50 eV (high). The leucine-enkephalin was used as the lock mass at *m*/*z* 554.2615. Data were acquired and processed using MassLynx 4.1 software (Waters).

### 2.3. Ethical Statement

All experimental procedures involving animals were approved by the Institutional Animal Care and Use Committee of Chonnam National University (CNU IACUC-YB-2025-73). All efforts were made to minimize animal suffering and the number of animals used.

### 2.4. Animals and Experimental Design

To evaluate the effects of AY BuOH in a DNCB-induced AD-like mouse, a DNCB-induced murine model was employed. Male SKH-1 hairless mice (6 weeks old, 20–25 g) were purchased from Samtako (Osan, Republic of Korea). Animals were acclimatized for one week under controlled conditions: temperature 22 ± 2 °C, relative humidity 60 ± 5%, and a 12 h light/dark cycle, with free access to standard rodent chow and water. After acclimatization, mice were randomly allocated into twelve groups (*n* = 5 per group): 1, Normal control (Mock + Vehicle); 2, AD-induced control (DNCB + Vehicle); 3, Positive control [DNCB + Oclacitinib (4 mg/kg/day)]; 4–12, AY BuOH-treated groups [2.5, 5, 10, 20, 40, 80, 160, 320, and 640 mg/kg/day].

AD-like skin inflammation was induced by topical application of 2,4-dinitrochlorobenzene (DNCB; Sigma-Aldrich, St. Louis, MO, USA). DNCB was dissolved in a mixture of acetone and olive oil (4:1, *v*/*v*). For initial sensitization, 1% DNCB solution (100 µL) was applied daily to the shaved dorsal skin for three consecutive days in all groups except the normal control. Subsequently, 0.5% DNCB solution (100 µL) was applied to the same area three times per week to maintain AD-like lesions throughout the 5-week experimental period.

During the induction period, animals received one of the following treatments by oral gavage once daily (7 times per week): vehicle control (0.5% CMC solution), Oclacitinib (4 mg/kg/day, positive control), or AY BuOH extract at the designated doses (2.5–640 mg/kg/day). Treatments were continued until the end of the experimental period.

### 2.5. Clinical Assessment (Dermatitis Severity Score; Modified SCORAD-like)

The severity of AD-like skin lesions was evaluated once weekly throughout the experimental period using a dermatitis severity score (modified SCORAD-like), adapted from previously established scoring approaches for experimental AD models [20,21]. Five clinical parameters were assessed on the dorsal lesional skin: erythema/hemorrhage, edema, excoriation/erosion, scaling/dryness, and lichenification. Each parameter was graded on a 4-point scale (0 = none, 1 = mild, 2 = moderate, 3 = severe), and the sum of the five subscores was calculated as the total dermatitis severity score (range: 0–15) [20]. Clinical scoring was performed independently by two observers blinded to group allocation, and the mean of the two scores was used for statistical analyses.

### 2.6. Serum Collection and IgE Measurement

At the end of the experiment (day 35), mice were anesthetized with isoflurane, and blood was collected from the jugular vein. Samples were centrifuged at 12,000× *g* for 5 min at 4 °C, and serum was stored at −80 °C until analysis. Total serum IgE concentrations were measured using a Mouse IgE ELISA kit (Thermo Fisher Scientific, Waltham, MA, USA), following the manufacturer’s instructions. Absorbance was measured at 450 nm using a microplate reader, and values were calculated against a standard curve.

### 2.7. Cytokine Analysis

Skin tissues (~100 mg) were homogenized in 1 mL T-PER tissue protein extraction reagent (Pierce, Rockford, IL, USA) containing a protease inhibitor cocktail. Homogenates were centrifuged at 12,000× *g* for 20 min at 4 °C, and the supernatants were stored at −80 °C. The concentrations of interleukin-4 (IL-4) and interleukin-13 (IL-13) were quantified using solid-phase sandwich ELISA kits (Thermo Fisher Scientific), according to the manufacturer’s instructions. A standard curve (0–500 pg/mL) was generated using four-parameter logistic regression, and concentrations were expressed as pg/mL.

### 2.8. Th1/Th2 Immune Profiling by Flow Cytometry

Th1 cells were defined as CD3^+^CD4^+^interferon-gamma-positive (IFN-γ^+^) cells, and Th2 cells were defined as CD3^+^CD4^+^IL-4^+^ cells. Spleens were aseptically removed and passed through a 70 μm cell strainer to prepare single-cell suspensions in DMEM (Welgen, Republic of Korea). After red blood cell lysis using ACK buffer, splenocytes were washed with PBS and adjusted to 1 × 10^6^ cells/mL. Cells were stimulated with Cell Stimulation MIX and Protein Transport Inhibitor MIX (Thermo Fisher Scientific) for 5 h at 37 °C in 5% CO_2_. After fixation and permeabilization, cells were stained with FITC-anti-CD4, PerCP-Cy5.5-anti-CD3, PE-anti-IFN-γ, and APC-anti-IL-4 antibodies. Flow cytometry analysis was performed using the Attune™ NxT Flow Cytometer (Thermo Fisher Scientific) under identical acquisition conditions, and data were analyzed with Attune™ NxT software v3.1.2. The percentages of Th1 (CD3^+^CD4^+^IFN-γ^+^) and Th2 (CD3^+^CD4^+^IL-4^+^) subsets were determined, and the Th1/Th2 ratio was calculated. The spleen index (%) was calculated as (spleen weight/body weight) × 100.

### 2.9. Histopathological Evaluation

At the end of the experiment, dorsal skin samples were fixed in 10% neutral buffered formalin, dehydrated through graded ethanol, embedded in paraffin, and sectioned at 4 μm thickness. Sections were deparaffinized with xylene, rehydrated, and stained with hematoxylin (1 min) and eosin (3 min). Histological evaluation included epidermal hyperplasia, hyperkeratosis, parakeratosis, spongiosis, dermal edema, and inflammatory cell infiltration, as previously described [22]. Mast cell infiltration was further evaluated using toluidine blue staining. Images were obtained using a light microscope (CX21, Olympus, Tokyo, Japan), and lesion severity was semi-quantitatively scored.

### 2.10. Statistical Analysis

All data were analyzed using GraphPad Prism software (version 9.5.1, GraphPad Software, San Diego, CA, USA). Results were expressed as mean ± standard deviation (SD). Statistical comparisons among groups were conducted using one-way analysis of variance (ANOVA) followed by Tukey–Kramer post hoc test. Assumptions of normality and homogeneity of variance were verified, and Welch’s ANOVA or nonparametric tests were applied when necessary. A *p*-value of <0.05 was considered statistically significant. Significance levels were indicated as follows: * *p* < 0.05, ** *p* < 0.01, ** *p* < 0.001, *** *p* < 0.0001.

## 3. Results

### 3.1. Identification of Compounds in the BuOH Fraction

**Figure 1:**
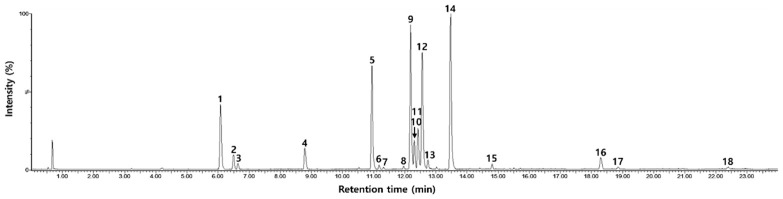
UPLC-ESI-QToF-MS/MS TIC chromatogram of the BuOH fraction.

The BuOH fraction of AY was analyzed using UPLC-ESI-QToF-MS/MS for qualitative profiling. As shown in the total ion current (TIC) chromatogram (Figure 1), eighteen compounds were detected in the BuOH fraction. The identification of the compounds was based on their accurate masses, retention times, UV absorbance, and MS/MS fragmentation patterns, as presented in Table 1. The compounds were all classified as either quercetin/kaempferol-type flavonoids or caffeic/ferulic acid-type hydroxycinnamic acid derivatives.

Compared to the results of the hot water extract, relatively polar compounds, including neochlorogenic acid (1), chlorogenic acid (2), caffeic acid (3), and feruloylquinic acid (4), exhibited higher peak intensities. In contrast, relatively nonpolar compounds, including feruloyl acetylrhamnoside (16, 17) and ethyferuloylquinic acid (18), showed lower peak intensities. Moreover, the isomeric diversity of malonyldicaffeoylquinic acid (13) and caffeoylferuloylquinic acid (15) was reduced, while dicaffeoylsuccinoylquinic acid and malonyldiferuloylquinic acid were not detected in the BuOH fraction. It is presumed that the reduction in nonpolar compounds resulted from their low solubility in BuOH during solvent fractionation. Nevertheless, rutin (5), 3,4-DCQA (9), 3,5-DCQA (12), and 4,5-DCQA (14) were still retained as the major compounds following solvent fractionation.

### 3.2. Quantitative Analysis of Major Compounds in the BuOH Fraction

Four major compounds identified in the BuOH fraction, including rutin, 3,4-DCQA, 3,5-DCQA, and 4,5-DCQA, were quantified using UPLC-ESI-TQ-MS/MS in MRM mode. As presented in Table 2, the calibration curves established using reference standards exhibited excellent linearity, which was demonstrated by the regression equations with coefficients of determination (R^2^) ranging from 0.9982 to 0.9999. The limits of detection (LOD) and quantification (LOQ) were calculated using the mean slope and the standard deviation of the y-intercept from the calibration curve to evaluate the sensitivity of the quantitative method. The LOD and LOQ were determined in the ranges of 0.01–0.03 μg/mL and 0.04–0.09 μg/mL, respectively, indicating the capability to detect and quantify analytes at trace levels. Based on the above data, quantitative analysis of the BuOH fraction revealed that 3,5-DCQA had the highest content at 137.56 ± 6.32 mg/g fraction, followed by 4,5-DCQA (121.02 ± 3.88 mg/g fraction), rutin (116.58 ± 6.25 mg/g fraction), and 3,4-DCQA (108.33 ± 2.98 mg/g fraction).

Previous studies reported that the rutin content in the 60% ethanol extract of AY was 3.60 μg/mg dried weight by HPLC analysis [16]. This result suggests that rutin was markedly enriched in the BuOH fraction, with its content approximately 32 times higher than that of the dried sample. It is presumed that this discrepancy results from differences in extraction solvent, solvent fractionation, and instrumental sensitivity. In contrast, the quantitative analysis of the three DCQA isomers was conducted for the first time in this study, despite having been previously identified in AY using NMR or LC-MS analyses [23].

### 3.3. Body Weight Changes

**Figure 2:**
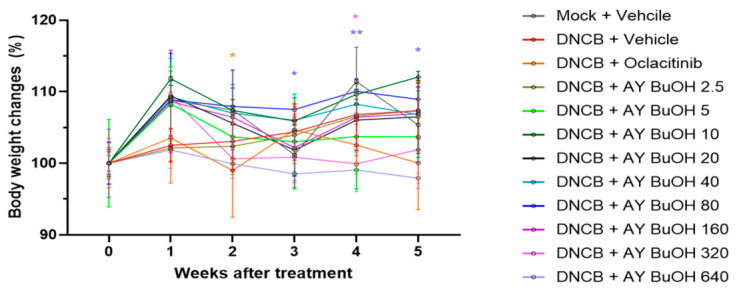
Body weight changes in mice over the 5-week experimental period. Groups included normal control (Mock + Vehicle), DNCB control (DNCB + Vehicle), Oclacitinib-treated (4 mg/kg/day), and AY BuOH extract-treated groups (2.5–640 mg/kg/day). Data are expressed as mean ± SD (*n* = 5 per group). * *p* < 0.05, ** *p* < 0.01 compared with the DNCB control group.

No significant differences in body weight were observed between the mock-treated control group (Mock + Vehicle) and the DNCB-treated control group (DNCB + Vehicle) throughout the 5-week experimental period (Figure 2). In the DNCB-induced groups, treatment with AY BuOH fractions at various doses (2.5–640 mg/kg/day) and Oclacitinib (4 mg/kg/day) was further assessed. Transient but non-sustained changes in body weight were observed in the Oclacitinib-treated group and in the BuOH-320 group. In contrast, the BuOH-640 group exhibited a continuous reduction in body weight, suggesting that 640 mg/kg/day may represent an upper tolerability boundary for AY BuOH administration.

### 3.4. Preliminary Hematological and Serum Biochemical Safety Screening

**Figure 3:**
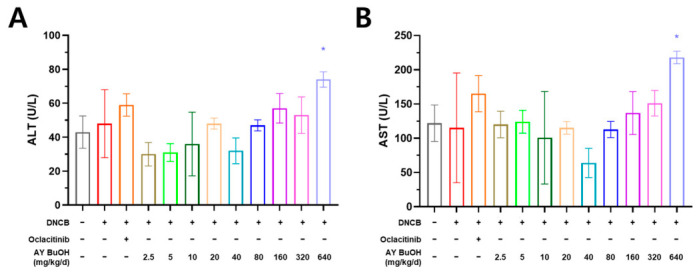
Serum alanine aminotransferase (ALT; (**A**)) and aspartate aminotransferase (AST; (**B**)) activities were measured as hepatic function markers. BUN and creatinine levels are provided in Supplementary Tables S1 and S2. Data are expressed as mean ± SD (*n* = 5 per group). * *p* < 0.05 compared with the normal control group.

To conduct a preliminary safety screening after repeated oral administration of AY BuOH, body weight changes, complete blood cell count parameters, and serum biochemical markers associated with hepatic and renal function were evaluated at the end of the experiment (Figure 3; Supplementary Tables S1 and S2). Serum alanine aminotransferase (ALT) and aspartate aminotransferase (AST) activities were significantly elevated in the BuOH-640 group compared with the normal control group, whereas no significant differences were observed in blood urea nitrogen (BUN) or creatinine levels among the experimental groups. Complete blood cell count parameters did not indicate marked hematological abnormalities under the present experimental conditions. These findings suggest potential hepatic stress at the highest dose, without clear hematological or renal biochemical evidence of systemic impairment. However, because this assessment did not include organ coefficients, gross necropsy, multi-organ histopathology, or toxicokinetic profiling, it should be interpreted as preliminary safety screening rather than a comprehensive in vivo safety evaluation.

### 3.5. Clinical Symptoms and SCORAD Assessment

**Figure 4:**
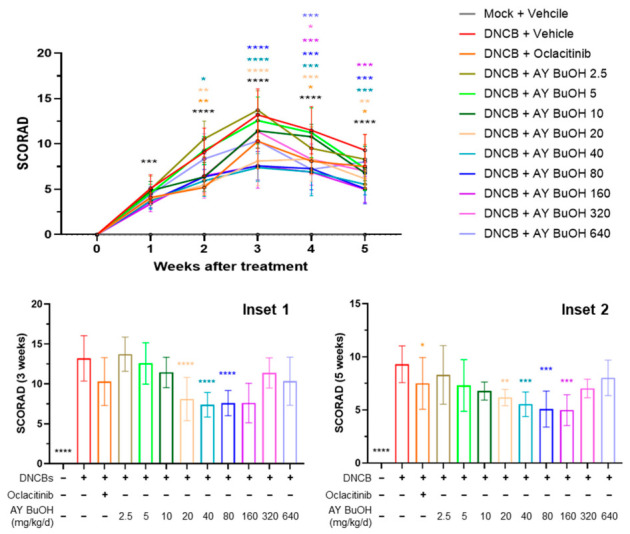
Modified dermatitis severity score evaluation in DNCB-induced AD-like mice treated with AY BuOH. Clinical severity was scored based on erythema, edema, excoriation, papulation, and lichenification. Inset panels show separate analyses at week 3 and week 5. Data are expressed as mean ± SD (*n* = 5 per group). * *p* < 0.05, ** *p* < 0.01, *** *p* < 0.001, **** *p* < 0.0001 compared with the DNCB control.

**Figure 5:**
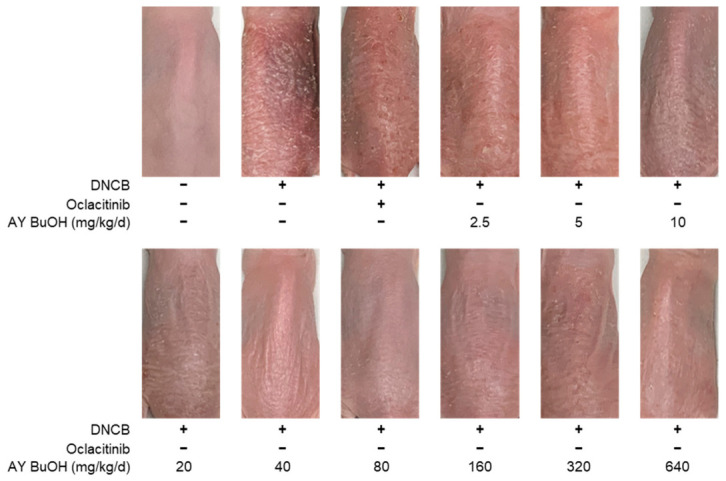
Representative gross skin lesions at week 5. Normal control, DNCB control, oclacitinib-treated, and AY BuOH-treated groups are shown. DNCB control mice exhibited erythema, edema, lichenification, and excoriation, whereas AY BuOH-treated groups displayed visible improvement in clinical symptoms.

Clinical severity of dermatitis was quantified using a modified dermatitis severity score, which incorporates erythema, edema, excoriation, papulation, and lichenification scores (Figure 4). DNCB-treated controls showed a significant and persistent increase in dermatitis severity scores from week 1 to week 5 compared with normal controls, confirming successful induction of AD-like lesions. Treatment with oclacitinib moderately reduced dermatitis severity scores, achieving statistical significance at weeks 2, 4, and 5. Notably, AY BuOH administration at doses ≥ 20 mg/kg/day resulted in consistent and significant improvements in dermatitis severity scores compared with DNCB controls. BuOH-20 and BuOH-40 reduced scores as early as week 2, while BuOH-80 showed improvements from week 3 onward. In comparison with the oclacitinib reference group, selected intermediate-dose AY BuOH groups showed comparable numerical improvements in dermatitis severity scores in this murine model. Representative gross skin lesions at week 5 demonstrated attenuation of erythema, edema, scaling, and lichenification in AY BuOH-treated mice (Figure 5).

### 3.6. Serum IgE Levels

**Figure 6:**
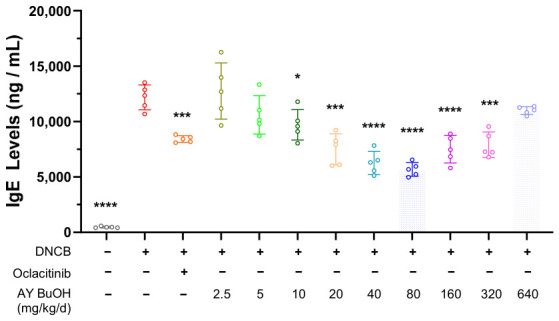
Effect of AY BuOH extract on serum IgE levels in DNCB-induced atopic dermatitis mice. Serum was collected at week 5 and analyzed by ELISA. Data are expressed as mean ± SD (*n* = 5 per group). * *p* < 0.05, *** *p* < 0.001, **** *p* < 0.0001 compared with the DNCB control.

Serum IgE concentrations were markedly increased in the DNCB-treated controls, consistent with systemic allergic sensitization (Figure 6). AY BuOH treatment at 20–320 mg/kg/day significantly reduced IgE levels compared with the DNCB group. Among the AY BuOH-treated groups, BuOH-40, BuOH-80, and BuOH-160 produced the most robust reductions in serum IgE levels compared with the DNCB control group.

### 3.7. Type 2 Inflammatory Cytokine Profiles

**Figure 7:**
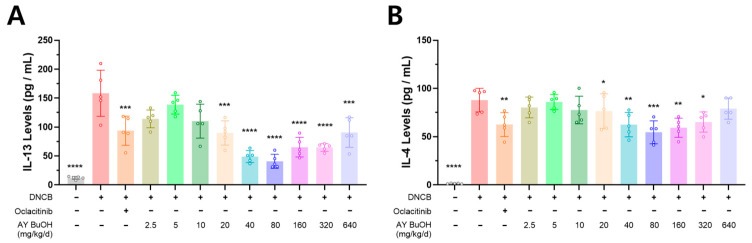
Effect of AY BuOH extract on inflammatory cytokine production in dorsal skin tissues. Cytokines IL-4 (**A**) and IL-13 (**B**) were quantified by ELISA at week 5. Data are expressed as mean ± SD (*n* = 5 per group). * *p* < 0.05, ** *p* < 0.01, *** *p* < 0.001, **** *p* < 0.0001 compared with the DNCB control.

Analysis of inflamed dorsal skin extracts revealed elevated IL-4 and IL-13 levels in DNCB-treated controls (Figure 7). AY BuOH treatment at doses ≥20 mg/kg/day significantly suppressed both cytokines, indicating attenuation of Th2-mediated inflammatory responses. The magnitude of suppression indicated robust attenuation of type 2 inflammatory responses in AY BuOH-treated groups.

### 3.8. Th1/Th2 Immune Balance

**Figure 8:**
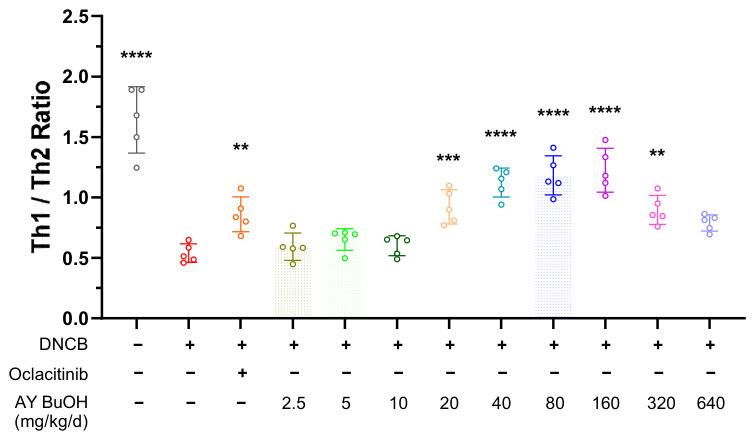
Flow cytometric analysis of Th1/Th2 balance in splenocytes from experimental mice. Th1 cells were defined as CD3^+^CD4^+^interferon-gamma-positive (IFN-γ^+^) and Th2 cells as CD3^+^CD4^+^IL-4^+^. Data are expressed as mean ± SD (*n* = 5 per group). ** *p* < 0.01, *** *p* < 0.001, **** *p* < 0.0001 compared with the DNCB control.

Flow cytometric analysis of splenocytes demonstrated a profound Th1/Th2 imbalance in DNCB-treated controls, characterized by reduced IFN-γ^+^ Th1 and increased IL-4^+^ Th2 populations (Figure 8). Almost all AY BuOH-treated groups exhibited significant restoration of Th1/Th2 ratios compared with DNCB controls. Particularly, BuOH-40, BuOH-80, and BuOH-160 showed marked restoration of Th1/Th2 balance compared with the DNCB control group.

### 3.9. Histopathological Findings

**Figure 9:**
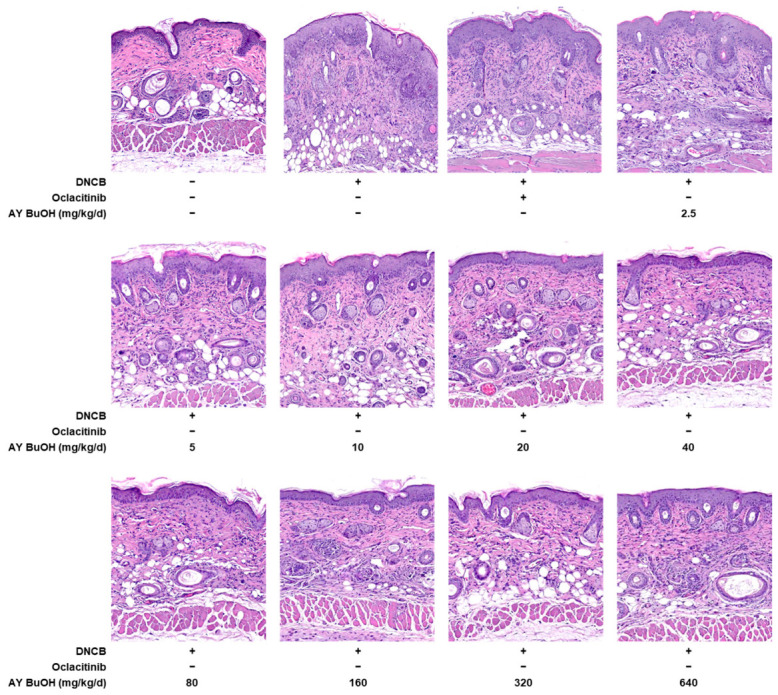
Representative histological sections of dorsal skin from experimental mice stained with H&E (100× magnification). DNCB control mice showed epidermal hyperplasia, dermal thickening, and chronic inflammatory changes. AY BuOH-treated groups demonstrated attenuation of pathological lesions.

**Figure 10:**
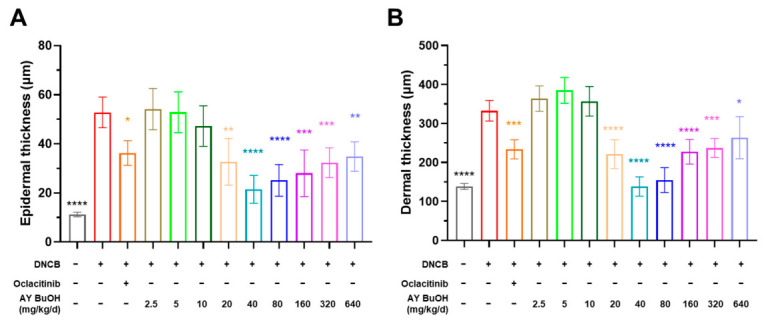
Quantitative histological analysis of (**A**) epidermal thickness and (**B**) dermal thickness in dorsal skin. Data are expressed as mean ± SD (*n* = 5 per group). * *p* < 0.05, ** *p* < 0.01, *** *p* < 0.001, **** *p* < 0.0001 compared with the DNCB control.

Histological analysis of dorsal skin sections stained with H&E revealed marked epidermal hyperplasia, hyperkeratosis, dermal thickening, and chronic inflammatory changes in DNCB-treated controls (Figure 9). AY BuOH treatment ameliorated these pathological features in an intermediate-dose-responsive manner, with significant reductions in epidermal and dermal thickness compared with DNCB controls (Figure 10). Notably, BuOH-40 and BuOH-80 showed prominent reductions in epidermal and dermal thickness compared with the DNCB control group.

## 4. Discussion

This study demonstrates that oral administration of AY BuOH attenuated DNCB-induced AD-like skin inflammation across multiple convergent endpoints, including SCORAD-like clinical scores, serum IgE, type 2-skewed cytokines (IL-4 and IL-13), splenic Th1/Th2 balance, and histopathological lesions. Rather than positioning AY BuOH as a pharmacological substitute for approved anti-pruritic drugs, these findings support its potential as a standardized plant-derived dietary supplement or functional feed additive candidate for atopic skin health. The DNCB-induced mouse model is widely used to reproduce major AD-like features, including type 2 immune activation, epidermal hyperplasia, inflammatory cell infiltration, and IgE-associated allergic responses [13]. Accordingly, the present results provide preclinical proof-of-concept evidence for further evaluation of AY BuOH as an orally administered functional ingredient for companion animal skin health.

The efficacy profile of AY BuOH appears to be related, at least in part, to its phytochemical composition. LC-MS/MS profiling and quantitative analysis showed that the BuOH fraction contained high levels of rutin and several DCQA isomers, including 3,4-DCQA, 3,5-DCQA, and 4,5-DCQA. The presence of quantifiable marker compounds is important for future feed-additive development because it supports standardization, batch-to-batch quality control, and dose consistency. DCQAs and caffeoylquinic acid derivatives have been reported to exert antioxidant and anti-inflammatory effects in skin-relevant experimental systems, while AY extract has been shown to suppress NF-κB-mediated inflammatory responses in vitro [15,24]. These findings provide a plausible chemical basis for the multi-endpoint improvements observed after AY BuOH administration, although bioactivity-guided fractionation will be needed to identify the principal active constituents and possible synergistic interactions.

The suppression of IL-4 and IL-13 by AY BuOH is particularly relevant to AD pathophysiology. These cytokines are central mediators of type 2 inflammation and contribute to IgE production, epidermal barrier impairment, pruritus, and chronic inflammatory remodeling [25,26]. In the present model, AY BuOH reduced IL-4 and IL-13 levels in inflamed dorsal skin and decreased serum IgE concentrations, indicating attenuation of both local and systemic allergic responses. These effects coincided with partial restoration of the splenic Th1/Th2 ratio, suggesting that AY BuOH may modulate systemic type 2 immune bias as well as local skin inflammation. This immunological profile is consistent with previous evidence that dietary polyphenols and flavonoids can regulate multiple immune-cell populations, cytokine networks, and inflammatory signaling pathways [27,28].

The clinical and gross lesion data further support the anti-inflammatory activity of AY BuOH. AY BuOH administration at intermediate doses, particularly 20–80 mg/kg/day, consistently improved SCORAD-like dermatitis scores and gross skin lesions compared with the DNCB control group. Because this study was conducted in mice, oclacitinib should be interpreted as a pharmacological reference control rather than as a basis for direct clinical comparison in dogs [6]. Thus, the responses observed in selected AY BuOH-treated groups indicate robust preclinical activity, but they do not establish clinical superiority over oclacitinib in target species.

Histopathological findings supported the clinical and immunological improvements observed after AY BuOH administration. DNCB-treated mice exhibited typical AD-like changes, including epidermal hyperplasia, hyperkeratosis, dermal thickening, and inflammatory cell infiltration, whereas AY BuOH, particularly at intermediate doses, attenuated epidermal and dermal thickening. These tissue-level changes indicate mitigation of AD-like inflammatory remodeling in the skin, consistent with the observed reductions in IgE and type 2 inflammatory cytokines.

The safety profile showed a dose-dependent pattern. Body weight loss and elevation of serum ALT and AST were observed mainly at 640 mg/kg/day, whereas BUN and creatinine did not differ significantly among groups. Complete blood cell count parameters did not indicate marked hematological abnormalities under the present experimental conditions. These findings suggest potential hepatic stress at the highest dose without clear hematological or renal biochemical evidence of systemic impairment. However, the present assessment did not include organ coefficients, gross necropsy, multi-organ histopathology, or toxicokinetic profiling; therefore, it should be interpreted as preliminary safety screening rather than a comprehensive repeated-dose toxicity study. Future studies should include organ weights, gross necropsy, multi-organ histopathology, toxicokinetic profiling, and target-species safety assessment before practical inclusion levels can be proposed for companion animal diets.

Oral administration was selected because AY BuOH was intended as a dietary supplement and functional feed additive candidate rather than as a topical dermatological drug. This route is consistent with companion animal studies evaluating nutritional supplements, complementary feeds, and functional diets as adjunctive strategies for chronic dermatological disorders, including canine atopic dermatitis [8,9]. Although this study did not assess pharmacokinetics, caffeoylquinic acid derivatives, including DCQAs, are reported to undergo extensive metabolism after oral intake, suggesting that orally generated metabolites may contribute to systemic biological activity [29,30,31]. The high-dose findings at 640 mg/kg/day further indicate the need for repeated-dose safety evaluation before target-species application.

A key translational issue is how the effective mouse dose range may inform future dog and cat studies. Direct mg/kg extrapolation from mice to companion animals is inappropriate because metabolic rate and body surface area differ across species. Body-surface-area-based interspecies dose conversion provides an initial framework, in which the target-species dose is calculated as target dose (mg/kg) = mouse dose (mg/kg) × (Km_mouse/Km_target) [32]. Using Km values of 3 for mice, 20 for dogs, and 13 for cats, the effective mouse dose range of 20–80 mg/kg/day corresponds approximately to 3–12 mg/kg/day in dogs and 4.6–18.5 mg/kg/day in cats. These converted values should be regarded only as preliminary estimates for designing target-species safety, pharmacokinetic, palatability, and pilot efficacy studies, not as recommended clinical or dietary doses. Practical dietary inclusion levels should be determined through future studies that consider formulation characteristics, marker-compound stability, gastrointestinal tolerance, and long-term safety.

These findings are consistent with the broader concept of nutritional and functional-feed approaches for canine dermatological disorders. Nutritional supplements and complementary feeds have been considered adjunctive tools in veterinary dermatology, particularly for chronic conditions requiring long-term management [9]. Recent evidence in dogs with AD further suggests that a functional diet containing a standardized plant-derived extract can improve clinical and skin barrier-related parameters, supporting the feasibility of diet-delivered functional ingredients for canine atopic skin disease [8]. In this context, AY BuOH is best positioned as a candidate ingredient for adjunctive skin-health support rather than as a stand-alone treatment for AD.

This study has several limitations. First, the DNCB-SKH-1 model is a chemically induced AD-like model and cannot fully recapitulate spontaneous canine or feline AD, which involves complex interactions among genetic predisposition, allergen exposure, skin barrier dysfunction, microbiome alterations, and chronic relapsing inflammation [33,34]. Second, the experimental period was limited to five weeks, and longer-term studies are needed to evaluate sustained efficacy and cumulative safety. Third, barrier function parameters such as transepidermal water loss, corneometry, skin hydration, and epidermal barrier protein expression were not directly evaluated, although they are important endpoints in companion animal AD [8,12]. Finally, although LC-MS/MS profiling identified candidate marker compounds, the active constituents responsible for the observed efficacy remain to be clarified.

Future studies should proceed through a stepwise translational framework. AY BuOH should first be standardized using major marker compounds such as rutin, 3,4-DCQA, 3,5-DCQA, and 4,5-DCQA, followed by validation of batch-to-batch consistency under feed-manufacturing conditions. Mechanistic studies should evaluate skin barrier markers, including filaggrin, loricrin, involucrin, and tight junction-associated proteins, together with type 2 signaling pathways such as IL-4/IL-13–STAT6 and NF-κB because type 2 cytokine signaling directly suppresses epidermal differentiation and barrier-related molecules in AD [35,36]. Subsequent target-species studies should assess palatability, digestibility, pharmacokinetics, long-term safety, and compatibility with commercial or hypoallergenic diets to determine whether AY BuOH can be practically incorporated into companion animal feeding regimens [8,37]. Ultimately, controlled feeding trials in dogs or cats with naturally occurring AD will be required to determine whether the preclinical immunomodulatory effects observed here translate into clinically meaningful improvements in pruritus, lesion scores, barrier function, and owner-reported outcomes.

In summary, AY BuOH, a fraction enriched in DCQAs and rutin, produced broad preclinical improvements in DNCB-induced AD-like dermatitis, including reduced clinical severity, lower serum IgE, suppression of IL-4 and IL-13, partial restoration of Th1/Th2 balance, and attenuation of histopathological lesions. The most consistent efficacy was observed at intermediate doses of 20–80 mg/kg/day, whereas the highest dose of 640 mg/kg/day was associated with body weight loss and hepatic enzyme elevation, indicating potential hepatic stress. Together, these findings support AY BuOH as a standardized plant-derived dietary supplement and functional feed additive candidate for companion animal skin health. However, target-species studies are required to define practical dosing, formulation suitability, long-term safety, and clinical efficacy in dogs and cats.

## 5. Conclusions

Oral administration of AY BuOH attenuated DNCB-induced AD-like skin inflammation in mice, with the most consistent responses observed at intermediate doses and potential hepatic stress detected at the highest dose. By integrating phytochemical characterization with clinical, immunological, histopathological, and preliminary biochemical safety outcomes, this study provides a rational preclinical basis for developing AY BuOH as a standardized plant-derived functional ingredient for atopic skin health. Further target-species studies are required to define practical dosing, formulation suitability, long-term safety, and clinical relevance in dogs or cats with naturally occurring atopic dermatitis.

## Figures and Tables

**Table 1:** Chemical library of the BuOH fraction.

No.	*t_R_* (min)	λ_max_ (nm)	Mode of Ionization	Measured Mass (Da)	Calculated Mass (Da)	Mass Error (ppm)	Molecular Formula	Fragment Ions (*m*/*z*)	Predicted Compounds
1	6.08	290, 325, 345	[M-H]^-^	353.0873	353.0873	0.0	C_16_H_18_O_9_	191.0554, 179.0341, 173.0456, 161.0235, 135.0442	Neochlorogenic acid
2	6.51	290, 325, 345	[M-H]^-^	353.0879	353.0873	1.7	C_16_H_18_O_9_	191.0565, 179.0346, 173.0458, 161.0248, 135.0446	Chlorogenic acid
3	6.63	290, 320, 340	[M-H]^-^	179.0343	179.0344	−0.6	C_9_H_8_O_4_	135.0443	Caffeic acid
4	8.79	290, 325, 345	[M-H]^-^	367.1029	367.1029	0.0	C_17_H_20_O_9_	193.0504, 191.0555, 173.0450, 134.0370	Feruloylquinic acid
5	10.94	265, 345	[M-H]^-^	609.1462	609.1456	1.0	C_27_H_30_O_16_	300.0280, 271.0253, 255.0299	Rutin
6	11.18	265, 345	[M-H]^-^	477.0674	477.0669	1.0	C_21_H_18_O_13_	301.0362, 271.0244, 255.0307	Miquelianin
7	11.32	265, 345	[M-H]^-^	463.0885	463.0877	1.7	C_21_H_20_O_12_	300.0286, 271.0248, 255.0303	Isoquercitrin
8	11.97	290, 325, 345	[M-H]^-^	515.1180	515.1190	−1.9	C_25_H_24_O_12_	353.0851, 335.0771, 191.0554, 179.0347, 173.0446, 161.0249, 135.0437	1,3-Dicaffeoylquinic acid
9	12.19	290, 325, 345	[M-H]^-^	515.1188	515.1190	−0.4	C_25_H_24_O_12_	353.0880, 335.0775, 191.0556, 179.0343, 173.0450, 161.0239, 135.0446	3,4-Dicaffeoylquinic acid
10	12.30	265, 345	[M-H]^-^	593.1511	593.1506	0.8	C_27_H_30_O_15_	285.0398, 255.0300, 227.0343	Nicotiflorin
11	12.42	290, 325, 345	[M-H]^-^	515.1188	515.1190	−0.4	C_25_H_24_O_12_	353.0870, 335.0763, 191.0556, 179.0340, 173.0448, 161.0237, 135.0447	3,5-*epi*-Dicaffeoylquinic acid
12	12.57	290, 325, 345	[M-H]^-^	515.1190	515.1190	0.0	C_25_H_24_O_12_	353.0878, 335.0771, 191.0557, 179.0344, 173.0451, 161.0238, 135.0446	3,5-Dicaffeoylquinic acid
13	12.75	290, 325, 345	[M-H]^-^	601.1205	601.1193	2.0	C_28_H_26_O_15_	557.1313, 515.1207, 395.0988, 353.0887, 233.0663, 191.0558, 179.0348	Malonyldicaffeoylquinic acid
14	13.48	290, 325, 345	[M-H]^-^	515.1193	515.1190	0.6	C_25_H_24_O_12_	353.0875, 335.0774, 191.0557, 179.0345, 173.0452, 161.0244, 135.0446	4,5-Dicaffeoylquinic acid
15	14.81	290, 325, 345	[M-H]^-^	529.1351	529.1346	0.9	C_26_H_26_O_12_	367.1044, 353.0884, 193.0509, 191.0558, 179.0351, 173.0455, 161.0251, 135.0449, 134.0370	Caffeoylferuloylquinic acid
16	18.30	290, 325, 345	[M-H]^-^	381.1187	381.1186	0.3	C_18_H_22_O_9_	193.0498, 134.0369	Feruloyl acetylrhamnoside
17	18.85	290, 325, 345	[M-H]^-^	381.1182	381.1186	−1.0	C_18_H_22_O_9_	193.0498, 134.0374	Feruloyl acetylrhamnoside
18	22.40	290, 325, 345	[M-H]^-^	395.1345	395.1342	0.8	C_19_H_24_O_9_	193.0505, 134.0372	Ethyferuloylquinic acid

**Table 2:** Regression equation, coefficient of determination (R^2^), LOD, and LOQ for rutin, 3,4-DCQA, 3,5-DCQA, and 4,5-DCQA in the BuOH fraction.

Compounds	Regression Equation	Coefficient of Determination (R^2^)	LOD (μg/mL)	LOQ (μg/mL)	Contents (mg/g Fraction, Mean ± SD, *n* = 3)
Rutin	y = 8713x − 34.26	0.9999	0.01	0.04	116.58 ± 6.25
3,4-DCQA	y = 21688x − 287.67	0.9994	0.02	0.06	108.33 ± 2.28
3,5-DCQA	y = 40314x − 412.67	0.9997	0.03	0.08	137.56 ± 6.32
4,5-DCQA	y = 40145x − 1455.40	0.9982	0.03	0.09	121.02 ± 3.88

## Data Availability

The data supporting the conclusions of this article are included within the article. Raw data are available from the corresponding author upon reasonable request.

## Supplementary Materials

The following supporting information can be downloaded at: https://www.mdpi.com/article/10.3390/ani16132003/s1, Table S1: Complete blood cell count (CBC) results; Table S2: Serum biochemistry results.
