# Supplementary material for: Oral Administration of Aster yomena Butanol Fraction Attenuates DNCB-Induced Atopic Dermatitis-like Skin Inflammation in Mice: Implications as a Dietary Supplement Candidate for Companion Animals
Source: Animals (Basel). 2026 Jun 30;16(13):2003. doi: 10.3390/ani16132003 (PMC13360054; doi:10.3390/ani16132003)
Supplement: Supplementary file 1 [file animals-16-02003-s001.zip › animals-4378950-supplementary.pdf]

Supplementary Materials

Supplementary Tables

Table S1. Complete blood cell count (CBC) results.

Parameter	AY BuOH (mg/kg/day)									
	NC	2.5	5	10	20	40	80	160	320	640
RBC (×10⁶/μL)	8.12 ± 0.32	8.45 ± 0.28	7.95 ± 0.30	8.53 ± 0.41	8.36 ± 0.25	8.45 ± 0.28	8.07 ± 0.41	8.27 ± 0.32	8.31±0.42	8.65 ± 0.40
HGB (g/dL)	10.21 ± 0.53	10.54 ± 0.38	11.02 ± 0.53	10.87 ± 0.36	11.21 ± 0.47	10.85 ± 0.62	10.57 ± 0.39	11.34 ± 0.54	11.21±0.33	10.89 ± 0.42
HCT (%)	38.35 ± 2.32	40.27 ± 2.85	37.38 ± 3.68	40.32 ± 2.64	38.32 ± 1.62	41.21 ± 2.38	37.37 ± 2.01	39.32 ± 2.44	40.21±2.82	39.67 ± 2.42
PLT (×10⁵/μL)	682 ± 20	712 ± 24	721 ± 18	694 ± 32	672 ± 27	698 ± 31	735 ± 31	725 ± 29	730±25	733 ± 37
WBC (×10³/μL)	4.92 ± 0.34	5.17 ± 0.42	4.96 ± 0.28	5.02 ± 0.27	4.57 ± 0.30	4.71 ± 0.21	4.47 ± 0.22	4.70 ± 0.24	4.51 ± 0.38	5.42 ± 0.42
Neutrophils (×10²/μL)	20.57 ± 2.52	20.14 ± 3.02	20.74 ± 3.14	17.33 ± 3.35	13.18 ± 4.21	14.95 ± 3.48	10.16 ± 3.25	15.19 ± 3.06	15.16 ± 2.42	20.49 ± 3.92
Lymphocytes (×10²/μL)	27.27 ± 3.24	30.08 ± 2.51	27.30 ± 2.77	31.41 ± 2.58	30.98 ± 2.88	30.56 ± 3.15	32.62 ± 2.89	30.07 ± 2.63	28.30 ± 3.78	32.27 ± 3.01
Monocytes (×10²/μL)	0.35 ± 0.07	0.37 ± 0.14	0.39 ± 0.11	0.38 ± 0.05	0.38 ± 0.09	0.36 ± 0.04	0.36 ± 0.08	0.35 ± 0.06	0.35 ± 0.14	0.38 ± 0.11
Eosionphils (×10²/μL)	0.82 ± 0.09	0.87 ± 0.19	0.96 ± 0.25	0.92 ± 0.14	0.96 ± 0.04	1.00 ± 0.13	1.07 ± 0.25	1.13 ± 0.17	1.05 ± 0.21	0.78 ± 0.12
Basophils (×10²/μL)	0.23 ± 0.12	0.23 ± 0.11	0.23 ± 0.05	0.19 ± 0.11	0.19 ± 0.08	0.20 ± 0.08	0.20 ± 0.04	0.22 ± 0.03	0.22 ± 0.04	0.24 ± 0.06

Note: NC, Normal control; WBC, RBC, Red blood cells; HGB, Hemoglobin; HCT, Hematocrit; PLT, Platelets; White blood cells; Statistically significant values of each index against those of NC were presented as *, p<0.05, **, p<0.01, and ***, p<0.001 respectively. The results were presented as mean ± S.D. (standard deviation)

Table S2. Serum biochemistry results.

Parameter	AY BuOH (mg/kg/day)									
	NC	2.5	5	10	20	40	80	160	320	640
Glu (mmol/L)	6.2 ± 0.5	7.2 ± 0.8	7.8 ± 0.2	6.7 ± 0.9	7.5 ± 0.5	7.7 ± 0.6	6.9 ± 0.4	6.8 ± 0.5	6.9 ± 0.3	7.4 ± 0.6
TAG (mmol/L)	2.5 ± 0.3	2.2 ± 0.4	2.5 ± 0.3	2.8 ± 0.3	2.1 ± 0.3	2.4 ± 0.3	2.7 ± 0.4	2.2 ± 0.4	2.5 ± 0.4	2.3 ± 0.3
Chol (mmol/L)	2.8 ± 0.4	3.8 ± 0.4	3.6 ± 0.4	3.2 ± 0.5	3.3 ± 0.4	2.6 ± 0.4	3.7 ± 0.3	3.5 ± 0.4	3.2 ± 0.2	3.7 ± 0.2
Crea (μmol/L)	10.2 ± 1.2	12.8 ± 1.5	10.8 ± 0.8	11.2 ± 1.5	13.2 ± 0.8	14.2 ± 2.1	13.4 ± 1.9	11.5 ± 1.7	14.2 ± 2.1	15.4 ± 2.5
BUN (mmol/L)	9.2 ± 1.2	10.5 ± 1.6	11.5 ± 2.2	8.3 ± 1.1	10.4 ± 1.6	11.7 ± 2.1	10.8 ± 1.7	11.2 ± 1.7	15.1 ± 2.4	15.7 ± 2.6
AST (U/L)	78 ± 5.1	99 ± 8.2	78 ± 8.4	89 ± 7.9	92 ± 9.2	86 ± 9.5	96 ± 10.1	85 ± 6.3	105 ± 8.2	145 ± 10.1 *
ALT (U/L)	58.2 ± 5.2	63.8 ± 7.1	72.1 ± 6.2	67.2 ± 5.8	72.2 ± 6.8	71.2 ± 7.7	65.7 ± 7.2	63.5 ± 8.2	69.2 ± 8.1	84 ± 9.7 *
ALP (U/L)	62.1 ± 3.4	78.3 ± 7.8	72.1 ± 5.4	75.5 ± 6.3	54.6 ± 5.2	48.3 ± 5.3	66 .7 ± 4.8	82.1 ± 6.2	75.8 ± 6.7	80.1 ± 5.8
Alb (g/L)	28 ± 3.1	34 ± 4.2	32 ± 4.3	25 ± 4.2	28 ± 3.4	31 ± 2.8	34 ± 4.1	35 ± 3.2	28 ± 4.5	30 ± 2.8

Note: Glu, Glucose; TAG, Triacylglycerol; Chol, Total cholesterol; Crea, Creatinine; BUN, Blood urea nitrogen; AST, Aspartate aminotransferase; ALT, Alanine aminotransferase; ALP, Alkaline phosphatase; Alb, Albumin. Data are presented as mean ± SD. *$p < 0.05$ compared with NC.
